# Common Ground of Medicine and Dentistry

**Published:** 1896-04

**Authors:** Joseph Roach

**Affiliations:** Baltimore, Md.


### Common Ground of Medicine and Dentistry.

Read in the Section on Dental and Oral Surgery, at the Forty-sixth Annual Meeting of the American Medical Association, at Baltimore, Md., May 7-10, 1895.

BY JOSEPH ROACH, M.D., BALTIMORE, MD.

At the outstart, definitions are necessary, and it is well to state that the term medicine includes all that the average practitioner would expect to do, who did not think of establishing himself in any one of the numberless so-called specialties of medicine. For convenience, I will call dentistry one of these specialties, although it might be shown that it is so distinct and so peculiar as to make it well-nigh a thing by itself and difficult to place with any of the specialties. If it be surgery, many operations consist in cutting out decayed parts in an organ so constituted that it has of itself no power to make use of the vis medicatrix naturæ, found in other organs and tissues, and with filling the same with some foreign material. The sole use of that material is to prevent further progress of the disease and to serve as a substitute for grinding and cutting food.

There is no blood, no ligation of arteries, no stitches, no sutures are made; and the patient is dismissed on the spot cured by a surgery, of which it might be said that its only relapses are due either to mechanical imperfections in the operations or to inherent faults in the structure repaired. True it is, that one department of this work trenches on surgery thus far; that in the exploration of the canals, occupying the roots of teeth, either by a failure to remove living tissue or a failure to disinfect any decomposed tissue, a local sepsis may occur, resulting in abscess and occasionally invoking grave consequences. But even this does not ordinarily rise higher in the scale of surgery than the treatment of a felon on the finger. I have thus purposely taken a low view of the daily work of dentistry for this reason. I think possibly too much pains have been taken by its membership to elevate a most useful, nay, a most indispensable calling, into a position to vie with surgery proper. I say daily work, for while it is true, that, as dentists, we are occasionally called upon to repair a fractured maxilla or even extirpate a tumor of the jaw, such operations are but occasional and in the nature of the case, it seems to me to fall as naturally into the domain of the general surgeon, as does the fracture of the femur. The true usefulness of the dental surgeon in such cases rests in his ability as mechanician to skillfully form and adjust special splints in case of fracture. This ability he forms from his daily work noticing artificial dentures or in his possessing special cutting instruments in the form of burs, etc., for the dental engine, instruments peculiarly adapted for his own work in removing decay from teeth and turned to occasional use as above in removing tumors. Still none the less skillful is the work of making plates, crowns, bridges, etc., where to the dexterity of the mechanic must be added the fine eye of the artist, restoring sunken features, harmonizing incongruities and above all and most perplexing of all, pleasing or failing to please the tastes of fading humanity.

But while all the above may be true, and the work of the average dental surgeon may be largely mechanical, the fact, that both the surgeon and the work on the human body, makes a bond of union between them which is worth study, inasmuch as whatever links in this bond are found to be the common property of both are of much greater interest than any dissimilar points.

Of the maladies of common interest I will mention those often obscure and occasionally grave diseases that involve the maxillary sinus. This is obvious, for the reason that in such cases the dental surgeon very often is apt to be the first observer on the ground. For whether we view these diseases as arising from diseased teeth or from some more remote and obscure cause, the dental surgeon, if he be ready to take up such cases for himself or turn them over to the surgeon, is none the less called on to diagnose accurately the symptoms, that they may be treated as purely from tooth-trouble or he may proceed on some other line. It is not in the province of this paper fully to describe all the various troubles that may arise in the maxillary sinus; but, if you will throw with me a rapid glance at its position and uses, it will be of service in following any line of treatment that may be suggested. It is situated above the so-called jaw teeth on either side of the upper jaw and extending from the first bicuspid (sometimes the cuspid) backward to the second molar of the same. Its size varies and its exterior or facial walls are quite thin, the palatal wall stronger and its floor very variable in shape and thickness. The only opening into it upward is into the nasal cavity. The situation of the cavity is surgically important, for when we consider the tendency of all mucous-membrane-covered surfaces to catarrhal troubles and the position of any cavity without natural drain, shut in by bony walls, and its only outlet narrow and pointing upward, we see at a glance how the difficulty or impossibility of any natural drainage and the tendency of fluids once accumulated could give even more trouble than does occur. Indeed, if we consider the tendency of catarrhal troubles to spread by continuity of surface, it seems a wonder that all cases of chronic nasal catarrh do not involve engorgement of the antrum itself. Certainly this is what one might expect. Yet it would seem that this is not only not true, but that, on the other hand, certainly from the partial view of the case taken by the dental surgeon, the lighting up of antral trouble can be traced often to diseases of the teeth, the roots of which lie under its floor.

The dissecting table reveals to the dental pathologist a curious state of affairs just here; but, in order to grasp this situation fully, it is necessary to state what comes much more closely under the observation of the dental surgeon, a fact that only the physiognomist studies as it should be, and that is, that in inheriting our tendencies, we inherit a mixture. Small jaws with large teeth are the rule, occasionally large jaws with small teeth; the back teeth of the father, the front teeth of the mother or vice versa. The jaws are sometimes so narrow and cramped and the teeth so large, that they crowd each other and such a lack of blood in the jaw, from non-use in mastication, makes bone and muscle without stamina. The result is that the large teeth of the upper maxilla, the roots being formed physiologically, by addition to their length are either forced through the floor of the antrum, or else, in the evolution of that chamber from six years to twenty-one, the periosteum fails to build thickly enough to cover those roots.

They are, however, always covered by the membranous lining of this cavity, else the vessels, passing into the foramina of these teeth, would be exposed to the atmosphere. All this is not likely to involve the antrum in pathologic trouble so long as the roots of these teeth are sound, but it may be fairly stated that more than one-half of the patients falling into the dentist's hands, sooner or later have pulpless molars, the roots of which project through the floor of the maxillary sinus and are objects more or less irritating to the tissues against which they rest. Again in the case of molars, the roots of which do not pass through the antral floor, in the case of devitalization, an ordinary alveolar abscess may occur with the sequelæ of absorption of the alveolar plate at the tip of the root and the outflowing of pus into the antrum itself. It is true that an abscess in such situation might not be of grave consequences, yet it is plainly a situation of risk. This may not often exist, for the reason that the widely-forked roots of the upper molars and their nearness to the outer walls of bone cause development of fistulous openings away from the antrum, yet it is a source of danger and as such should be studied. Dentists devitalize and remove the pulp from the roots of such teeth. This is a very usual, almost daily, operation. But while an earnest and faithful effort is made to perform this operation thoroughly, so attenuated are the canals of the molars, that is not at all certain that all the pulp is removed. In such a case it is easy to see that any filling material forced into the root canal may force particles of dead tissue through the foramen and into the tissues above, inoculating these with septic matter; or, if disinfectants have been used there, such as carbolic acid, corrosive sublimate, etc., may be forced through to the injury of a tissue which has, as described in the outset, no drainage in case of chronic irritation. I have said enough on this subject, I dare say, to call your attention to certain lesions of the antrum, that are common ground of observation and treatment of both dentist and surgeon, and call attention in conclusion to a case, sometimes very grave, always painful, that of an impacted and abscessed third lower molar.

The same causes to which I have already alluded, as dwarfing the upper jaw, conjoin to make the lower both small and short. In the evolution of the lower wisdom tooth, it seems less often to share in the dwarfing process than the corresponding upper tooth. In a word, it is often too large for the space between the second molar and the angle of the jaw. In consequence its eruption is so interfered with, that it may be either only partially erupted or it may be impacted at an angle against the second molar, appearing through the gum slightly or not at all. Abscess of this tooth often involves not only the tissues immediately around it, but the tissues of the throat become inflamed and grave results ensue. It is pretty well established that throat trouble of a serious, or even fatal character, has been set up by an agency seemingly so unimportant. Abscess, opening into the pharynx, is certainly one of the sequelæ of this trouble. I have been told of cases, where the infiltration of pus between the muscles of the neck and connective tissue proceeded to the formation of fistulous openings on the stomach with ordinary results of blood poisoning.

I have said enough to indicate a common ground for dentistry and surgery in the two cases above cited. Many more might be mentioned, if time permitted, to strengthen the statement thereof.

